# Discovery of novel quinazoline-sulfonamide derivatives with promising antidiabetic activity

**DOI:** 10.3389/fchem.2026.1800775

**Published:** 2026-04-29

**Authors:** Mohamed F. Zayed, Yosra Muhammad, Aymen A. Alqurain, Mohammed A. A. Banafa, Abdelsattar M. Omar

**Affiliations:** 1 Pharmaceutical Sciences Department, Fakeeh College for Medical Sciences, Fakeeh Care Group, Jeddah, Saudi Arabia; 2 Department of Pharmaceutical Chemistry, Faculty of Pharmacy, King Abdulaziz University, Jeddah, Saudi Arabia; 3 Department of Clinical Practice, Faculty of Pharmacy, Northern Border University, Rafha, Saudi Arabia; 4 Doctor of Pharmacy Program, Fakeeh College for Medical Sciences, Fakeeh Care Group, Jeddah, Saudi Arabia

**Keywords:** antidiabetic, design, development, quinazoline-sulfonamide, synthesis

## Abstract

**Introduction:**

Five new quinazoline-sulfonamide hybrids 4a (MZ-13), 4b (MZ-20), 4c (MZ-25), 4d (MZ-26), and 6a (MZ-29) were designed, synthesized, and investigated for their *in vitro* and *in vivo* antidiabetic activities.

**Methods:**

The *in vivo* screening was conducted in a mouse model of type II diabetes induced by streptozotocin (STZ), using glibenclamide as the positive control. The in vitro model was performed by measuring the activity of these compounds against the PPARγ enzyme. Furthermore, the total antioxidant capacity (TAC) was measured for these compounds to assess their ability to neutralize a wide range of free radicals. A physicochemical study was conducted to demonstrate the drugability of these compounds. Additionally, the *in silico* ADMET and toxicity studies illustrated good pharmacokinetic properties and a low toxicity profile. Likewise, a comprehensive molecular modeling study was performed to examine the binding modes of the new compounds.

**Results:**

Compound MZ-29 showed 27.1% reduction in blood glucose (BG) levels, and the standard glibenclamide showed 17.2% reduction in BG. The in vitro assay of the compounds MZ-13 and MZ-29 demonstrated superior or comparable activity to the reference glibenclamide.

**Discussion:**

The study identified MZ-29 and MZ-26 as the most promising candidates in the series. These two compounds achieved docking scores and binding orientations closely mimicking the native ligand.

## 1 Introduction

Type 2 diabetes (T2D) is a chronic condition that results in elevated BG levels due to the body’s inadequate use of insulin (Gómez-Peralta et al., 2024; Schmidt, 2018). It is the predominant kind of diabetes that impacts millions of individuals globally (Zayed and Awis, 2024). While it is more prevalent among older people, it also impacts children and adolescents as a result of childhood obesity (Magkos et al., 2020; Zayed et al., 2025). Dietary management and exercise with oral hypoglycemics are the primary treatment for T2D to regulate BG levels and prevent diabetic complications (Bodmer et al., 2008). Numerous categories of oral hypoglycemic medications exist, including sulfonylureas, biguanides, meglitinides, thiazolidinediones, dipeptidyl peptidase-4 (DPP-4) inhibitors, glucagon-like peptide-1 (GLP-1) receptor agonists, and sodium-glucose co-transporter-2 (SGLT2) inhibitors (Fuchtenbusch et al., 2000; Fukuen et al., 2005). Sulfonylureas are recognized cornerstone treatments for T2D (Saczewski et al., 2010). The second-generation sulfonylurea glibenclamide is extensively utilized globally (El-Zahabi et al., 2022). This medication functions by enhancing insulin production from pancreatic β-cells (Suaifan et al., 2015). It interacts with sulfonylurea receptors (SUR) and inhibits ATP-sensitive K^+^ (KATP) channels in pancreatic β-cells to promote insulin production (Ibrahim et al., 2017a). This process is triggered by both low and high BG levels, resulting in hypoglycemia (Panchal et al., 2018). Moreover, it possesses numerous negative effects like weight gain, hypoglycemia, and GIT problems (Bodmer et al., 2008). Consequently, medicines that enhance insulin production in response to elevated glucose levels with minimal side effects will be highly valued.

**FIGURE 1:**
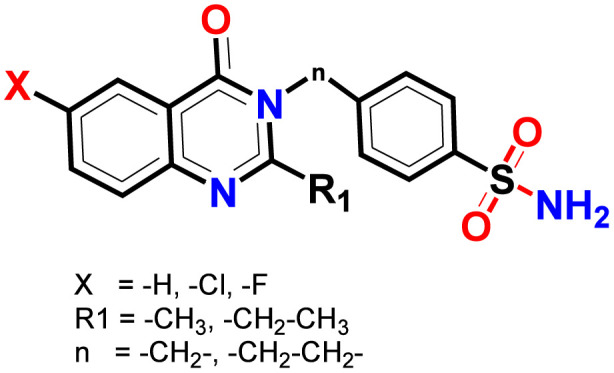
General model of the newly synthesized compounds.

Many quinazoline derivatives were reported as potent antihyperglycemic agents (Zayed, 2014; Zayed et al., 2015; Zayed et al., 2018). In our previous study, we presented a series of quinazoline-sulfonamide hybrids as analogs of sulfonyl urea (El-Zahabi et al., 2022). These compounds were evaluated for their antidiabetic efficacy and demonstrated encouraging results. As part of our ongoing research, we developed new derivatives that retain the same structural framework with alterations, specifically the incorporation of halo substitutions (fluoro and chloro) at the C6 of the quinazoline. Figure 1 shows the general model of our newly synthesized compounds.

### 1.1 Study rationale

**FIGURE 2:**
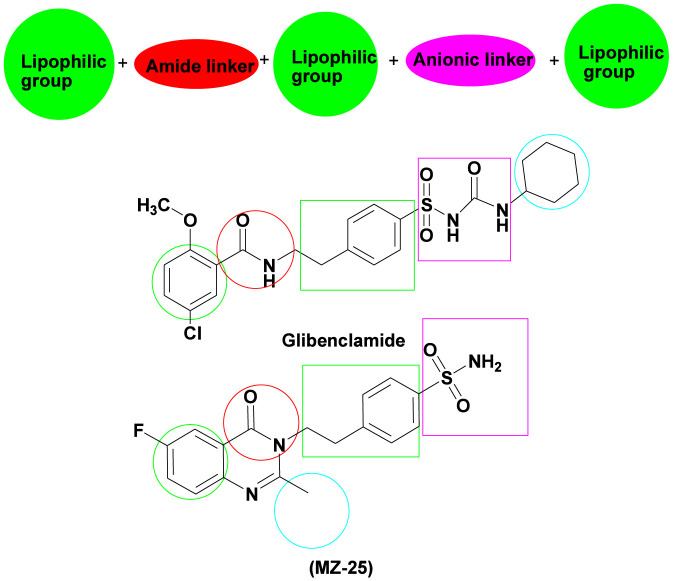
The structural similarities between glibenclamide and compound MZ-25.

The target compounds were designed based on the structural similarity with the antidiabetic sulfonylurea glibenclamide. Figure 2 displays the structural similarity between compound MZ-25 and glibenclamide.

It is worth noting that these compounds differ from our previously synthesized derivatives in that they contain halo substitution in the aromatic ring with a chloride or fluoride atom at C6 of the quinazoline system. This alteration is predicated on the latter reasons.

1. Electronic modulation and improving binding to the sulfonylurea receptors (SUR1).
2. Optimization of lipophilicity and membrane permeability
3. Blocking metabolic oxidation.
4. Steric and conformational impact.
5. Potential for advanced selectivity and low side effects.

#### 1.1.1 Electronic modulation and improving binding to the sulfonylurea receptors (SUR1)

Glibenclamide and newly synthesized derivatives have structural similarities and exhibit their antidiabetic activity by binding to the SUR1 subunit of the pancreatic β-cells (KATP) channel. These receptors are sensitive to polarity and electronic distribution of the ligand. Both fluoride and chloride are electron-withdrawing groups (EWG) due to their high electronegativity (El-Zahabi et al., 2022). Substitution at the 6th position of the quinazoline ring induces electron-deficient properties in the aromatic system. This process can favorably alter the electronic complementarity between target compounds and the key amino acid residue in the binding pocket of SUR1, like phenylalanine and tyrosine. The altered electronic environment, including dipole moment and electrostatic potential, can strengthen the π-π stacking or dipole-dipole interactions, leading to higher binding affinity and potency (Ibrahim et al., 2017b).

#### 1.1.2 Optimization of lipophilicity and membrane permeability

Optimal antidiabetic activity requires a balance between hydrophilicity and lipophilicity. Chloro-substitution with a chloride atom produces moderate lipophilicity, increasing the overall log P (partition coefficient) of the molecule, which can enhance passive diffusion across the β-cell membrane to reach the binding site in SUR1 (Bajare et al., 2012). Additionally, it can improve the general pharmacokinetic properties, like absorption and distribution. Fluoro-substitution, the fluoride atom has a small atomic size and high electronegativity, which can sometimes lead to polar hydrophilic interactions that improve metabolic stability without drastically changing log P. The fluoro-aromatic interaction is a common stabilizing force in protein-ligand binding. Consequently, using chloro and fluoro substitutions allows us to improve the lipophilicity profile for potency and bioavailability (Saczewski et al., 2010).

#### 1.1.3 Inhibition of metabolic oxidation

Unsubstituted or electron-rich aromatic rings at ortho or para positions, like that of glibenclamide, are primary targets for metabolic enzymes, cytochrome P450 (CYP-mediated oxidation). Substitution of the aromatic ring with chloride or fluoride blocks this process. Fluoride is an effective blocker of oxidative metabolism due to the strength of the C-F bond, which is resistant to CYP-mediated cleavage. It effectively slows down the hydroxylation of the aromatic ring. Chlorine also deactivates the ring toward oxidation and occupies a potential site of phase I metabolism. Overall, this modification process improves metabolic stability, plasma half-life, and antidiabetic potency (Fukuen et al., 2005).

#### 1.1.4 Steric and conformational effect

The presence of small atoms such as fluoride or chloride can influence the conformation of the quinazoline ring or the orientation of adjacent pharmacophoric groups like the sulfonamide side chain. This optimized conformation may present the sulfonamide moiety more precisely to its key hydrogen bonding partners on SUR1 (Gampe et al., 2000).

#### 1.1.5 Potential for advanced selectivity and low side effects

**FIGURE 3:**
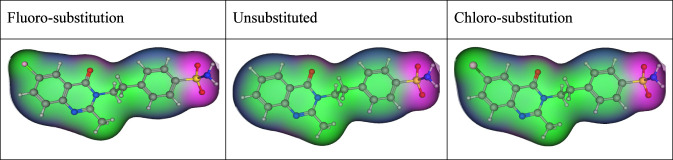
The electrostatic potential maps for fluoro-substituted, unsubstituted, and chloro-substituted quinazoline-sulfonamide hybrid (MZ-25)

Enhanced affinity for the pancreatic SUR1 over cardiac SUR2A is a needed goal to minimize cardiovascular risks. Electronic and steric effects caused by 6-halo substitution could affect binding to SUR1, potentially improving the therapeutic index. However, this requires experimental validation (Ibrahim et al., 2017a). Figure 3 displays the electrostatic potential maps for fluoro-substituted, unsubstituted, and chloro-substituted quinazoline-sulfonamide hybrid (MZ-25), showing the electronic modulation between them.

## 2 Results and discussion

### 2.1 Chemistry

**SCHEME 1:**
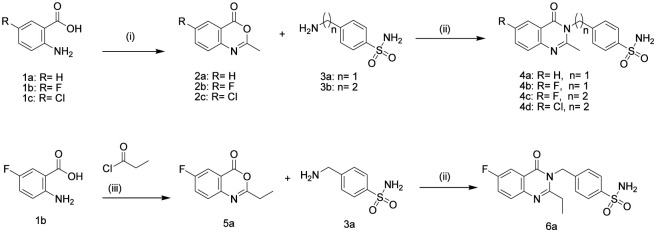
(i) acetic anhydride, reflux 1–3 h; (ii) glacial acetic acid, sodium acetate, reflux 24 h; (iii) dry pyridine, RT, 2 h.

The synthetic process of our novel compounds, 4a (MZ-13), 4b (MZ-20), 4c (MZ-25), 4d (MZ-26), and 6a (MZ-29), was accomplished through two reactions as explained in Scheme 1. The first reaction involves cyclodehydration of the appropriately substituted anthranilic acid. It proceeds via a nucleophilic acyl substitution pathway, followed by an intramolecular cyclization. The product of this reaction (benzoxazinone) is formed by refluxing substituted anthranilic acid with acetic anhydride or acid chloride for 1–3 h. The second reaction includes nucleophilic ring-opening followed by Tandem cyclocondensation. The primary sulfonamide in this reaction acts as a nucleophile, attacking the electrophilic carbonyl carbon (C4) of the benzoxazinone, leading to ring opening followed by cyclization and aromatization to form final products (quinazoline-sulfonamide). Scheme 1 displays the synthetic pathway of our newly synthesized compounds.

### 2.2 Physicochemical features

The physicochemical properties of the target compounds support their feasibility as therapeutic agents. These molecules adhere to the Lipinski rule of five, exhibit a favorable bioavailability profile, demonstrate feasible synthetic accessibility, display high GIT absorption, and a low toxicity profile (http://www.swissadme.ch/). These characteristics indicate strong potential for development into orally administered drugs. Table 1 shows the physicochemical characteristics of the target compounds (Swiss Institute of Bioinformatics, 2025).

**TABLE 1:** Physicochemical characteristics of the target compounds.

Feature	MZ-13 (4a)	MZ-20 (4d)	MZ-25 (4c)	MZ-26 (4b)	MZ-29 (6a)
Structure	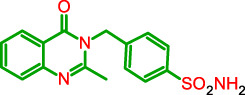	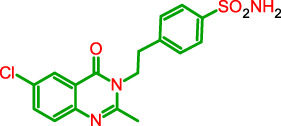	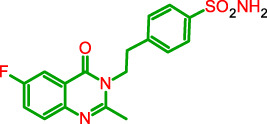	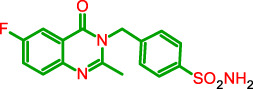	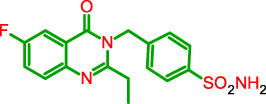
Formula	C_16_H_15_N_3_O_3_S	C_17_H_16_ClN_3_O_3_S	C_17_H_16_FN_3_O_3_S	C_16_H_14_FN_3_O_3_S	C_17_H_16_FN_3_O_3_S
Molecular weight	329.37 g/mol	377.85 g/mol	361.39 g/mol	374.36 g/mol	361.39 g/mol
Hydration free energy	−11.19	−9.82	−9.61	−10.29	−8.63
Boiling point	445.16	472.81	446.71	437.06	444.87
pKa acidic	7.69	7.81	7.22	6.87	7.02
pKa basic	1.98	2.12	2.27	1.83	2.19
Log (P)	1.62	2.42	2.23	2.00	2.32
Log (S)	−3.34	−4.27	−3.64	​	−3.42
Log Pa (vapor pressure)	−8.43	−9.08	−8.64	−8.52	−8.63
Heavy atoms	23	25	25	24	25
aromatic heavy atoms	16	16	16	16	16
Fraction Csp3	0.12	0.18	0.18	0.12	0.18
Rotatable bonds	3	4	4	3	4
H-bond acceptors	5	5	6	6	6
H-bond donors	1	1	1	1	1
Molar refractivity	87.71	97.53	92.48	87.67	92.48
TPSA	103.43 A^2^	103.43 A^2^	103.43 A^2^	103.43 A^2^	103.43 A^2^
Water solubility	Moderate	Poor	Moderate	Moderate	Moderate
GIT absorption	High	High	High	High	High
BBB permeant	No	No	No	No	No
Skin permeation	No	No	No	No	No
Drug likeness	Yes	Yes	Yes	Yes	Yes
Lipiniski rule	No violation	No violation	No violation	No violation	No violation
Synthetic accessibility	2.42	2.55	2.58	2.49	2.68

### 2.3 SAR of quinazoline derivatives in antidiabetic activity

Structure-activity relationships of antidiabetic quinazolines were reported in several papers. They are affected by many factors, including hydrophobicity, electron-withdrawing groups, hybridization, and substitution with selected groups (Abou-Seri et al., 2019). Increased hydrophobic parameters (e.g., log p) correlate with the higher potency. Substitution with electron-withdrawing groups such as halogens or nitro groups at key positions enhances activity by modulating electronic properties (Pham et al., 2013). Combining quinazoline with thiazolidinedione, oxadiazole, or sulfonamide moieties yields dual-acting antidiabetic agents. Substitution with some groups like Trichloromethyl, methyl, and anilino is essential for the activity and modulates the binding process. Substitution with Azabicyclic amines, phenyl, and carboxamido enhances GPR119 agonism and enzyme inhibition. Substitution with dimethoxy, dialkoxy boosts PPARγ binding, overall potency (Ibrahim et al., 2025).

### 2.4 Biology

#### 2.4.1 *In vivo* antidiabetic screening

The antidiabetic screening was conducted following the reported method (El-Zahabi et al., 2022; Ibrahim et al., 2017a; Ramsey et al., 2007). The first step is the induction of diabetes and baseline measurement. The second step is the treatment phase (2 mg/kg for 6 days). It includes administration of a dose (2 mg/kg) of the test compound daily for 6 days and measuring BG at the end of this period. Glibenclamide was used as a standard drug in the positive control group. The negative control group was used for comparing measurements.

##### 2.4.1.1 After induction (baseline diabetes confirmation)

All treatment groups showed significantly elevated glucose levels compared to negative controls after STZ induction (ANOVA: F = 4.8308, p = 0.000204***), confirming successful diabetes induction. Table 2 displays the BG levels and p-values after induction of diabetes.

**TABLE 2:** The compounds, BG levels, and p-values after induction of diabetes.

Compound	Mean ± SD	% Elevation	p-value vs. negative control	Order
MZ-13	373.75 ± 42.31	206.9	0.000567***	4
MZ-20	383.33 ± 127.12	214.7	0.069256	3
MZ-25	474.75 ± 98.58	289.9	0.005067**	1
MZ-26	299.33 ± 66.26	145.8	0.039615*	6
MZ-29	385.67 ± 91.63	216.6	0.035968*	2
Glibenclamide	358.00 ± 160.87	193.9	0.060119	5
Negative control	121.80 ± 15.53	0	-	-

Values are displayed as mean ± SD, or number, *P < 0.05, **P < 0.01, ***P < 0.001.

##### 2.4.1.2 After drug treatment (2 mg/kg)

**FIGURE 4:**
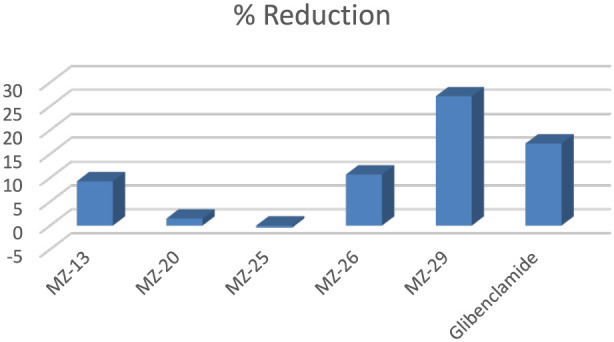
The compounds and their % reduction of BG compared to the reference glibenclamide after drug treatment (2 mg/kg). The dispersion values are 42.48%, 53.23%, 20.99%, 32.96%, 34.83%, 40.18% for the compounds MZ-13, MZ-20, MZ-25, MZ-26, MZ-29, and Glibenclamide respectively. Data are presented as the mean ± SD.

The tested dose was 2 mg/kg following the reported procedures (El-Zahabi et al., 2022; Ibrahim et al., 2017b). Compounds 13, 26, and 29 showed significant glucose reduction compared to the negative control group. Compound MZ-29 showed higher glucose reduction (27%, 2 mg/kg) than the standard (17.2%, 2 mg/kg). Compounds MZ-13, MZ-20, MZ-25, and MZ-26 showed lower glucose reduction activity than the standard. Compound MZ-25 showed significantly higher glucose than glibenclamide (p = 0.046). The order of activity of these compounds at 2 mg/kg was MZ-29, Glibenclamide, MZ-26, MZ-13, MZ-20, and MZ-25, respectively. The overall statistical analysis was: (ANOVA: F = 2.4434, p = 0.017376 *). Table 3 displays the compounds, BG levels, p-values, and activity order. Figure 4 shows the compounds and their % reduction of BG.

**TABLE 3:** The compounds, BG levels, and p-values after drug treatment (2 mg/kg).

Compound	Mean ± SD	% Reduction	p-value vs. neg. Control	p-value vs. glibenclamide	Order
MZ-13	339.0 ± 144.01	9.3	0.029232*	0.644858 (ns)	4
MZ-20	377.75 ± 201.08	1.5	0.086588 (ns)	0.521426 (ns)	5
MZ-25	476.50 ± 100.03	−0.4	0.005841 (ns)	0.046161*	6
MZ-26	267.20 ± 88.08	10.7	0.022387*	0.648910 (ns)	3
MZ-29	281.00 ± 97.88	27.1	0.023366*	0.803956 (ns)	1
Glibenclamide	298.80 ± 120.06	17.2	0.031667*	-	2
Negative control	125.00 ± 5.70	-	-	-	-

Values are displayed as mean ± SD, or number, *P < 0.05, **P < 0.01, ***P < 0.001.

#### 2.4.2 *In vitro* antidiabetic screening

##### 2.4.2.1 PPARγ agonistic assay

**FIGURE 5:**
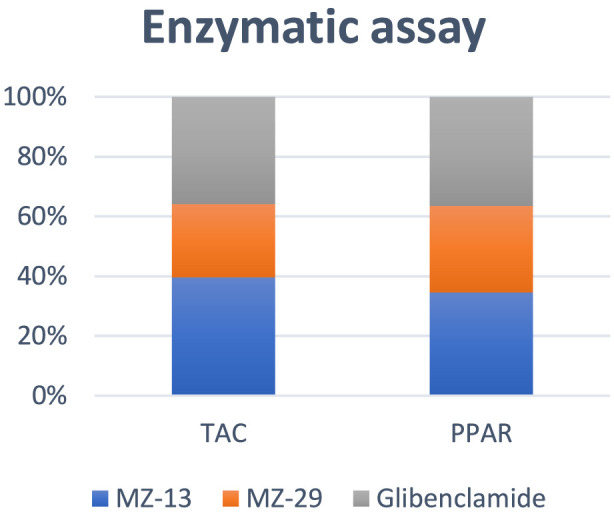
Enzymatic assay of MZ-13, MZ-29, and glibenclamide against PPARγ and TAC. The dispersion values are 38.57%, 67.69%, 0.15%, and 0.10% for MZ-13 and MZ-29 in TAC and PPAR assays, respectively. Data are presented as the mean ± SD of three independent experiments.

It measures the ability of the compounds to modulate the PPARγ pathway by measuring agonistic activity. PPARγ is a receptor responsible for the regulation of genes involved in glucose homeostasis, lipid metabolism, and anti-inflammatory responses. Drugs that are full PPARγ agonists are classified as a distinctive antidiabetic class, like thiazolidinedione. They are potent insulin sensitizers and have strong antidiabetic activity. This test was conducted using the reported method (Ibrahim et al., 2017b). MZ-13 and MZ-29 had activity of 370 pg/mL and 310 pg/mL, respectively. Glibenclamide had an activity of 390 pg/mL. MZ-13 demonstrated PPARγ pathway activity nearly equivalent to glibenclamide. Table 4 and Figure 5 show the PPARγ agonistic activity of compounds MZ-13 and MZ-29 compared to the reference glibenclamide.

**TABLE 4:** Results of the enzymatic assays of compounds MZ-13, MZ-29, and standard glibenclamide. The data provided are presented as mean ± SD.

Assay	Mean ± SD (MZ-13)	Mean ± SD (MZ-29)	Mean ± SD (glibenclamide)
TAC (nmol/mL)	2.1 ± 0.81	1.3 ± 0.88	1.9 ± 1.07
PPARγ (pg/mL)	370 ± 0.54	310 ± 0.31	390 ± 0.90

##### 2.4.2.2 Total antioxidant capacity (TAC)

This assay evaluates the non-specific cumulative antioxidant power of the compound. It measures the ability to neutralize a wide range of free radicals like DPPH, ABTS, and proxy radicals, or to reduce oxidants. It was conducted using the reported method (Silvestrini et al., 2023). The result is expressed in ascorbic acid equivalents. It answers the question of how much oxidative stress this compound can quench, regardless of the specific mechanism. Compounds MZ-13 and MZ-29 showed TAC of 2.1 nmol/mL and 1.3 nmol/mL, respectively. The standard glibenclamide showed TAC of 1.9 nmol/mL. MZ-13 displayed a superior TAC compared to glibenclamide. MZ-29 displayed lower TAC compared to MZ-13 and glibenclamide. Table 4 and Figure 5 show the total antioxidant capacity of compounds MZ-13 and MZ-29 compared to the reference glibenclamide.

### 2.5 Study limitations and future directions

This study presents significant evidence for the antihyperglycemic properties of the novel quinazoline-sulfonylurea hybrids, specifically MZ-29 and MZ-26; however, several limitations must be recognized. First, plasma insulin levels were not assessed, which would offer direct evidence of the compounds' impact on insulin secretion or sensitivity. Second, the study used a single-dose screening method instead of full dose-response curves. Third, the molecular mechanisms responsible for the observed antihyperglycemic effects have yet to be completely clarified. Future research is intended to overcome these limitations and to provide comprehensive mechanistic analyses of the most promising compounds, MZ-29 and MZ-26.

### 2.6 Molecular modeling

#### 2.6.1 Docking and induced-fit binding modes

All five MZ compounds, the reference ligands rosiglitazone and glibenclamide, successfully docked into the PPARγ ligand-binding site according to the reported procedures (Aljahdali et al., 2024; Mohamed et al., 2023; Omar et al., 2023). Initial Glide SP docking scores ranged from approximately −11.1 to −12.1 for the MZ series, indicating high shape and chemical complementarity to the binding pocket, comparable to the native agonist rosiglitazone (docking score −12.3) and significantly better than glibenclamide (−10.8) (Table 5).

**TABLE 5:** Docking and binding energy metrics for rosiglitazone, glibenclamide, and the MZ compounds docked into PPARγ (PDB ID: 1FM6), including Glide SP scores, MM-GBSA ΔG_bind, Prime energy, and IFDScore. More negative values indicate more favorable predicted binding and pose stability.

Title	Docking score	Glide e model	Glide g score	MMGBSA dG bind	Prime energy	IFD score
Rosiglitazone	−12.315	−92.313	−12.32	−79.31	−9269.59	−475.8
Glibenclamide	−10.847	−84.033	−10.874	28.54	−9291.28	−475.44
MZ-13	−11.119	−86.94	−11.119	−46.65	−9246.91	−473.46
MZ-20	−11.413	−79.966	−11.413	−17.48	−9222.35	−472.53
MZ-25	−11.668	−88.943	−11.669	−6.49	−9218.17	−472.58
MZ-26	−11.597	−81.231	−11.597	−47.94	−9255.84	−474.39
MZ-29	−12.056	−87.021	−12.057	−48.59	−9264.27	−475.27

The subsequent Induced Fit Docking refinement allowed key side chains (e.g., from residues Gln275, Phe313, Arg316, and Ser289) to adjust and accommodate each ligand. The top-ranked IFD poses revealed a conserved binding mode across the series: all ligands engage PPARγ′s polar activation region via at least one strong hydrogen bond to Gln275, and settle in a hydrophobic cavity lined by Phe313 and neighboring residues—notably, the IFD Score rankings aligned with known ligand characteristics. Rosiglitazone (native agonist) yielded the most favorable IFD score (−475.8) and served as a benchmark for an optimal binding mode. Among the new compounds, MZ-29 and MZ-26 achieved IFD scores (−475.3 and −474.4, respectively) nearly as favorable as rosiglitazone, whereas MZ-25 and MZ-20 had slightly higher (less negative) IFD scores (≈−472.5), indicating suboptimal interactions relative to the others. Glibenclamide, interestingly, obtained a very favorable IFD score (−475.4) close to that of the potent agonists, suggesting that the induced-fit protocol was able to find a pose where glibenclamide snugly fits the pocket. However, as discussed below, the apparent good docking of glibenclamide did not translate to binding stability or favorable energetics in subsequent analyses.

**FIGURE 6:**
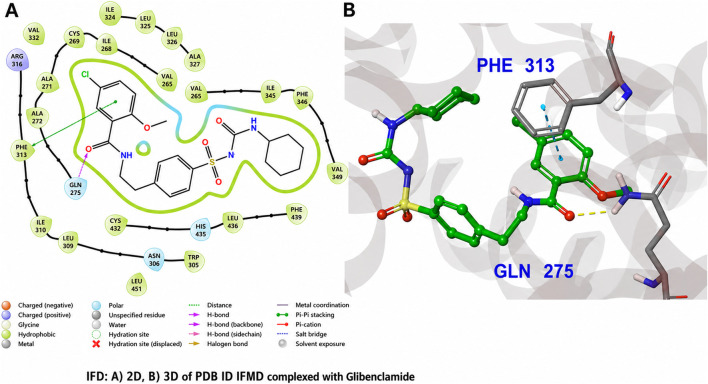
Induced Fit Docking (IFD) pose of glibenclamide in the PPARγ ligand-binding domain (PDB ID: 1FM6). **(A)** 2D interaction diagram illustrating hydrogen bonding between the sulfonylurea moiety of glibenclamide and Gln275 and Ala327 (magenta dashed lines), along with a π–π stacking interaction with Phe313. **(B)** 3D binding pose of glibenclamide (green sticks) within the PPARγ pocket, highlighting key surrounding residues. Glibenclamide occupies the entrance region of the binding site, with its chlorophenyl ring oriented toward Phe313 and the cyclohexyl group accommodated in a hydrophobic sub-pocket, while lacking interactions with the deeper activation helix region (e.g., His323 and Tyr473), consistent with a non-classical or partial agonist binding mode.

**FIGURE 7:**
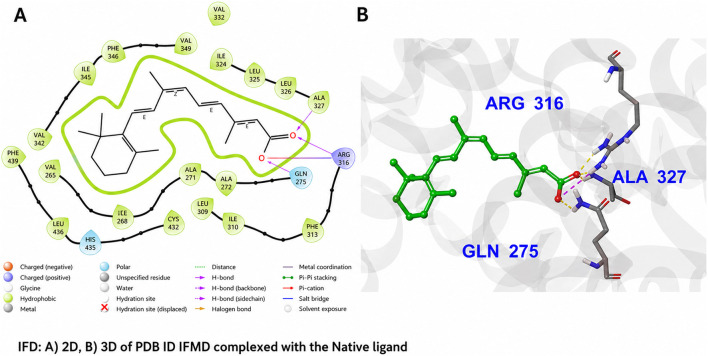
Induced Fit Docking (IFD) pose of the native agonist rosiglitazone in the PPARγ ligand-binding domain (PDB ID: 1FM6). **(A)** 2D interaction diagram illustrating hydrogen-bond interactions of the thiazolidinedione moiety with Gln275 and Ser289, along with additional polar contacts involving the pyridine nitrogen. Canonical interactions with His323 and Tyr473, characteristic of full agonist binding, are preserved in the induced-fit model but are not explicitly depicted in the 2D schematic. **(B)** 3D binding pose of rosiglitazone (magenta sticks) showing deep insertion into the ligand-binding pocket and extension toward helix H12, enabling formation of the conserved hydrogen-bond network associated with receptor activation. Hydrophobic and polar contacts with Phe313 and Arg316, respectively, further stabilize the bound conformation. The predicted pose closely recapitulates the experimentally observed binding mode (El-Zahabi et al., 2022).

In the induced-fit docking pose of glibenclamide (Figures 6A,B), the sulfonylurea moiety establishes hydrogen-bond interactions with Gln275 and the backbone carbonyl of Ala327 via the urea NH group, anchoring the ligand near the entrance of the PPARγ ligand-binding pocket. The hydrophobic chlorobenzamide and cyclohexyl substituents are accommodated within a predominantly nonpolar sub-pocket, forming van der Waals contacts with Phe313 and Leu330, while a π–π stacking interaction is observed between the benzylic phenyl ring of glibenclamide and Phe313, with an approximately parallel ring orientation (∼4.5 Å separation). Despite these stabilizing contacts, glibenclamide does not extend into the deeper activation region of the pocket and lacks interactions with His323 and Tyr473 on helix H12, residues that are critical for full PPARγ agonist activation. This relatively shallow binding mode is consistent with the reported weak or partial agonistic activity of glibenclamide toward PPARγ (El-Zahabi et al., 2022; Sherman et al., 2006). By contrast, rosiglitazone in its co-crystallized pose, following induced-fit refinement, penetrates more deeply into the PPARγ ligand-binding pocket (Figures 7A,B). The thiazolidinedione head group forms a bidentate hydrogen-bond network with His449 (corresponding to His323 in alternative PPARγ numbering schemes) and Tyr473 on helix H12, interactions that stabilize the active receptor conformation and are characteristic of full agonist binding (El-Zahabi et al., 2022; Scheen, 2007). In the induced-fit model, rosiglitazone additionally retains a hydrogen bond to Gln275 via its pyridinyl nitrogen and an interaction with Ser289, while its aromatic scaffold engages in hydrophobic packing against Phe313. The simultaneous engagement of anchoring residues at the pocket entrance and the activation helix region provides a structural basis for the high binding affinity and full agonist behaviour of rosiglitazone toward PPARγ.

**FIGURE 8:**
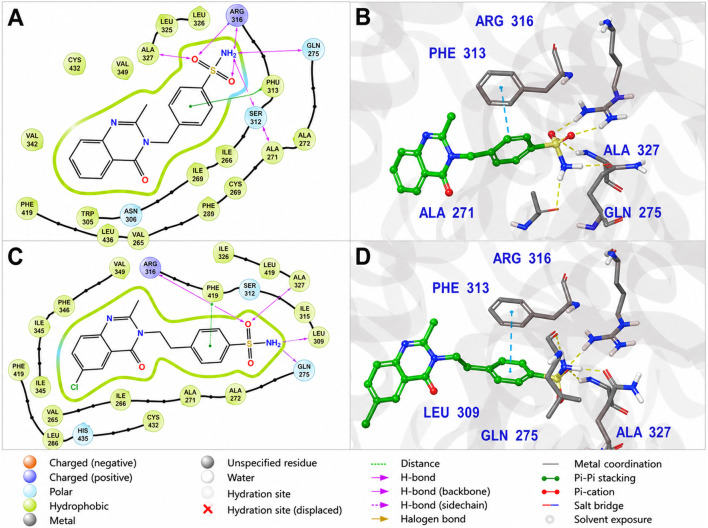
Induced Fit Docking (IFD) binding modes of MZ-13 and MZ-20 in the PPARγ ligand-binding domain (PDB ID: 1FM6). **(A)** 2D interaction diagram of MZ-13 showing conserved hydrogen-bond interactions with Gln275 and Ala327, together with a water-mediated hydrogen bond to Ser289. Aromatic π–π stacking with Phe313 contributes to the stabilization of the ligand at the pocket entrance. **(B)** 3D binding pose of MZ-13 (orange sticks), illustrating stable accommodation near the entrance of the binding site with partial extension toward the inner cavity, enabling interaction with residues proximal to Ser289. **(C)** 2D interaction diagram of MZ-20 highlighting the same conserved hydrogen-bonding pattern to Gln275 and Ala327, accompanied by increased hydrophobic contacts arising from its larger aromatic scaffold. **(D)** 3D binding pose of MZ-20 (cyan sticks) showing deeper occupation of the hydrophobic pocket and local side-chain rearrangements (notably Phe313) to accommodate the dichlorophenyl substituent. Despite improved hydrophobic packing, MZ-20 does not engage residues in the activation helix (H12) region, consistent with its lower predicted binding affinity relative to more optimally positioned analogues.

The novel MZ ligands adopted broadly similar binding orientations, each anchored by a conserved hydrogen bond to Gln275. For clarity, two representative examples from the series are discussed. MZ-13, one of the smaller analogues, assumes a binding pose that substantially overlaps with that of rosiglitazone. As shown in Figures 8A,B, MZ-13 forms a strong hydrogen bond with Gln275 via its sulfonylurea NH group, together with an additional backbone hydrogen bond to Ala327. Its aromatic scaffold occupies the same hydrophobic sub-pocket as the benzamide ring of glibenclamide, engaging in π–π stacking with Phe313, while a polar substituent is oriented toward Ser289, enabling a water-mediated interaction. Collectively, this interaction pattern secures the ligand at the pocket entrance and mid-cavity region but does not extend into the activation helix (H12) region, consistent with a binding mode more characteristic of partial agonists. MZ-20, by contrast, is a larger and more hydrophobic derivative. As illustrated in Figures 8C,D, MZ-20 preserves the conserved hydrogen bonds to Gln275 and Ala327, but its bulkier aromatic substituents penetrate further into the hydrophobic interior of the binding pocket. This results in additional van der Waals contacts with Cys285 and Ile326, with the distal phenyl ring positioned proximal to Met364. However, this deeper hydrophobic engagement is not accompanied by productive interactions with the activation helix, as MZ-20 does not establish contacts with His323 or Tyr473. The induced-fit model further indicates local side-chain rearrangements, particularly involving Phe313 and Met364, to accommodate the increased steric demand of MZ-20. This suboptimal balance between hydrophobic packing and polar anchoring likely underlies the slightly less favorable IFD score and binding free energy of MZ-20 relative to MZ-13 and the top-ranking analogues.

**FIGURE 9:**
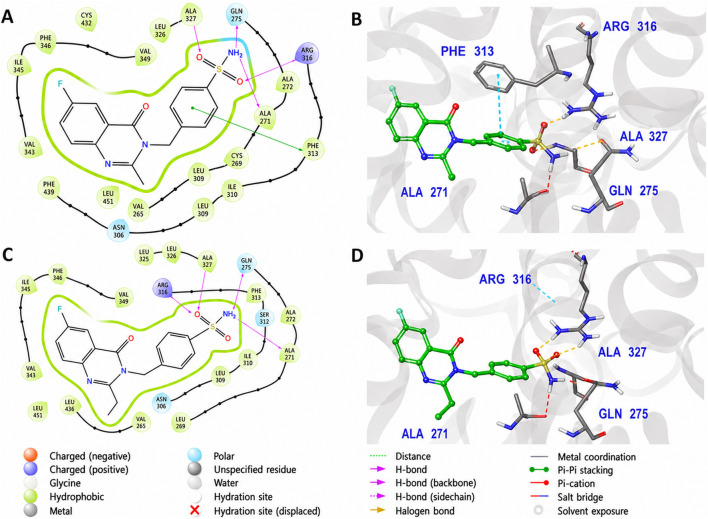
Induced Fit Docking (IFD) binding modes of MZ-26 and MZ-29 in the PPARγ ligand-binding domain (PDB ID: 1FM6). **(A)** 2D interaction diagram of MZ-26 illustrating dual hydrogen-bond interactions between the sulfonylurea NH groups and Gln275 and the backbone of Ala327, together with π–π stacking against Phe313. A polar carbonyl group is oriented toward His323, within potential hydrogen-bonding distance. **(B)** 3D binding pose of MZ-26 (pink sticks) showing efficient occupation of the hydrophobic pocket and favorable orientation toward the activation region. **(C)** 2D interaction diagram of MZ-29 highlighting conserved hydrogen bonding to Gln275 and Ala327, along with an additional interaction between the carboxylate substituent and Ser289. **(D)** 3D binding pose of MZ-29 (teal sticks) demonstrating deep pocket engagement, extensive hydrophobic and aromatic contacts, and local side-chain adjustments (notably Arg316) that accommodate the bulky polycyclic scaffold. The well-engaged binding mode is consistent with the high predicted affinity of MZ-29.

The two best-performing new ligands were MZ-26 and MZ-29, both of which achieved docking scores and induced-fit binding modes comparable to the native agonist rosiglitazone. MZ-26 (Figures 9A,B) contains a sulfonylurea linker analogous to that of glibenclamide and two aromatic rings that efficiently occupy the hydrophobic core of the PPARγ ligand-binding pocket. In its induced-fit pose, MZ-26 establishes a bifurcated hydrogen-bonding network: one sulfonylurea NH interacts with Gln275, while the second NH forms a backbone hydrogen bond with Ala327. In addition, the para-substituted phenyl ring lies in a favorable orientation for π–π stacking with Phe313, maximizing aromatic stabilization. Notably, MZ-26 positions a polar carbonyl oxygen toward His323, approaching hydrogen-bonding distance (∼3.2 Å), suggesting partial mimicry of the polar head-group interactions characteristic of full PPARγ agonists. MZ-29, the largest analogue in the series, exhibited the most favorable MM-GBSA binding free energy and a highly optimized induced-fit pose (Figures 9C,D). MZ-29 maintains robust anchoring via hydrogen bonding to Gln275 and forms an additional stabilizing interaction with Ser289, enabled by the introduction of a carboxylate substituent. Its extended polycyclic aromatic scaffold occupies a hydrophobic groove lined by Ile281 and Met364, generating extensive van der Waals contacts. Despite its increased steric bulk, MZ-29 is accommodated without unfavorable clashes; the induced-fit protocol captures local side-chain rearrangements, particularly involving Arg316, which shifts modestly to create space for the ligand core. This favorable adaptation is reflected in MZ-29’s highly competitive IFD score and binding free energy.

Collectively, the docking and induced-fit analyses indicate that all MZ compounds engage the canonical PPARγ pocket through a conserved anchoring interaction with Gln275. However, subtle differences in pocket penetration and engagement of the inner activation region—particularly proximity to His323 and Tyr473—distinguish the highest-affinity ligands (MZ-26 and MZ-29) from weaker analogues (MZ-20 and MZ-25). By contrast, glibenclamide, while capable of binding near the pocket entrance, lacks key interactions with the activation helix and is therefore consistent with its reported weak or partial PPARγ agonist activity (Ibrahim et al., 2017b).

#### 2.6.2 Binding affinity estimation

The Prime MM-GBSA binding free energy calculations provided further insight into the relative ligand affinities (Table 5). Rosiglitazone (native ligand) was predicted to bind very strongly to PPARγ (ΔGbind ≈ −79 kcal/mol), consistent with it being a high-potency full agonist. Among the test compounds, MZ-29, MZ-26, and MZ-13 showed the most favorable ΔGbind values (approximately −49, −48, and −47 kcal/mol, respectively). This suggests that these three compounds form sufficiently extensive interactions with the receptor to approach the binding energetics of rosiglitazone. MZ-29 in particular stands out; its large size and multiple contact points likely translate into a greater desolvation and interaction energy upon binding, hence the top-ranked ΔGbind. In contrast, MZ-20 and MZ-25 had substantially less favorable binding energies (−17.5 and −6.5 kcal/mol, respectively), indicating weaker binding. The much higher (less negative) ΔGbind for MZ-25 implies that it may not effectively stabilize the receptor–ligand complex, aligning with its less optimal induced-fit pose (MZ-25 did not engage the critical Phe313 or H12 region strongly). Glibenclamide’s MM-GBSA result was markedly unfavorable (+28.5 kcal/mol), suggesting that in the context of the full protein and solvent, glibenclamide binding is not thermodynamically favored. A positive ΔGbind implies that glibenclamide’s desolvation cost and induced strain outweigh the enthalpic gains from binding–an outcome consistent with the fact that glibenclamide is not an optimized PPARγ ligand and only a very weak partial agonist in biological assays (Magkos et al., 2020). We note that MM-GBSA tends to exaggerate absolute magnitudes, but the relative ranking here correlates with the qualitative docking findings: MZ-29 ≈ MZ-26 > MZ-13 >> MZ-20 > MZ-25 >> glibenclamide. This ranking was later supported by the dynamic stability of the complexes, as described next.

#### 2.6.3 Molecular dynamics (MD) simulation

**FIGURE 10:**
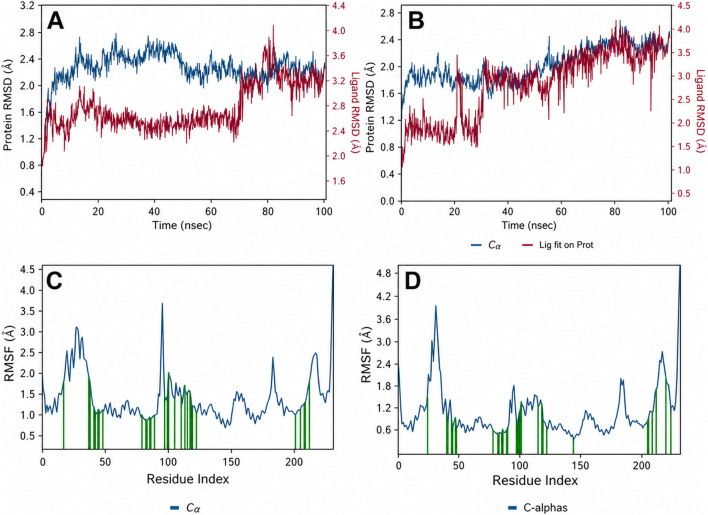
Molecular dynamics (MD) stability and flexibility of reference PPARγ complexes with glibenclamide and the native agonist rosiglitazone. **(A)** Time evolution of protein Cα RMSD (blue) and ligand RMSD (magenta) for the glibenclamide–PPARγ complex over 100 ns, showing stable protein behaviour but a pronounced increase in ligand RMSD at later time points, indicative of reduced binding stability. **(C)** Corresponding residue-wise RMSF profile of PPARγ in complex with glibenclamide, highlighting increased flexibility in regions proximal to the ligand-binding pocket. **(B)** Protein and ligand RMSD profiles for the rosiglitazone–PPARγ complex, demonstrating sustained ligand stability and limited conformational drift throughout the simulation. **(D)** RMSF profile of PPARγ in the presence of rosiglitazone, showing comparatively reduced fluctuations within the ligand-binding region, consistent with a well-stabilized agonist-bound conformation.

To validate the docking poses and assess complex stability under dynamic conditions, 100 ns MD simulations were performed for each protein–ligand complex. All systems reached equilibration within approximately 10 ns and remained structurally stable thereafter, with protein backbone RMSD values fluctuating within ∼2–3 Å relative to the minimized starting structures. Analysis of ligand RMSD profiles revealed clear differences in binding stability between the reference complexes (Figures 10A,B). In the glibenclamide–PPARγ complex (Figure 10A), the ligand heavy-atom RMSD remained within ∼1.5–2.0 Å during the first ∼75 ns, consistent with a metastable pose closely resembling the docked conformation. However, during the final ∼25 ns, the RMSD increased sharply to approximately 4 Å, indicating a pronounced rearrangement of the ligand within the binding pocket. Trajectory inspection showed that glibenclamide progressively drifted toward the pocket entrance and lost its stabilizing hydrogen bond with Gln275 after ∼80 ns. This reduction in dynamic stability is consistent with its unfavorable ΔGbind and the absence of strong anchoring interactions within the inner activation region. By contrast, the native agonist rosiglitazone exhibited markedly greater dynamic stability throughout the simulation (Figure 10B). The ligand RMSD remained below ∼2 Å for the majority of the trajectory, with a gradual increase to ∼3–4 Å toward the end of the 100 ns run. This modest rise likely reflects a limited reorientation of the thiazolidinedione head group while remaining bound, rather than ligand disengagement. RMSF profile of PPARγ in complex with glibenclamide is flexible (Figure 10C). Importantly, rosiglitazone retained its key stabilizing interactions with His323 and Gln275 for approximately 80% of the simulation time, maintaining the agonist-bound conformation of helix H12, as also supported by the reduced residue-level flexibility observed in the corresponding RMSF profile (Figure 10D).

**FIGURE 11:**
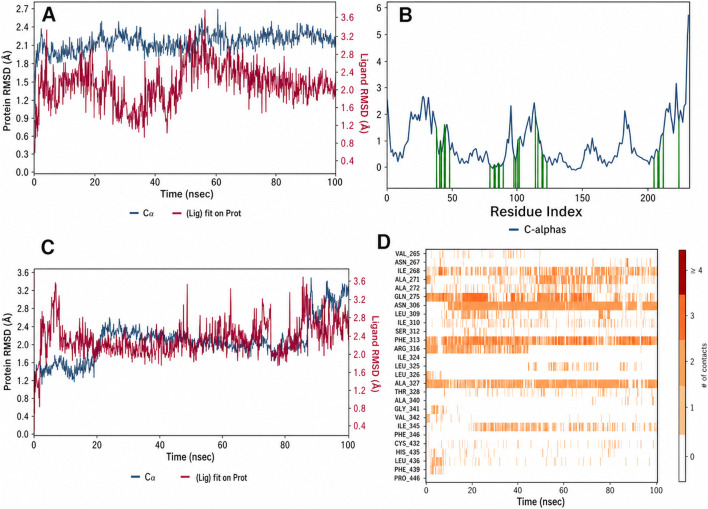
Molecular dynamics (MD) simulation analysis of MZ-13 and MZ-20 complexes with PPARγ. **(A)** Time evolution of protein Cα RMSD (blue) and ligand RMSD (red) for the PPARγ–MZ-13 complex over 100 ns, showing stable protein behaviour (≈2–2.5 Å) and low ligand RMSD (≈1–2 Å), indicative of a well-maintained binding pose. **(B)** Residue-wise RMSF profile of PPARγ in complex with MZ-13, highlighting increased flexibility in surface-exposed loop regions, while residues within the ligand-binding pocket (approximately residues 270–330) remain comparatively rigid, consistent with effective pocket stabilization by MZ-13. **(C)** RMSD profiles for the PPARγ–MZ-20 complex, where the ligand RMSD increases gradually from ∼2 Å to ∼3–3.5 Å toward the end of the simulation, indicating reduced dynamic stability relative to MZ-13, while the protein backbone remains stable. **(D)** Protein–ligand contact timeline for MZ-20, showing intermittent loss of key interactions, particularly involving Gln275 and Phe313, at later stages of the trajectory. These contact disruptions correlate with the increased ligand RMSD observed in panel **(C)**, suggesting a less persistent binding mode for MZ-20 under dynamic conditions.

**FIGURE 12:**
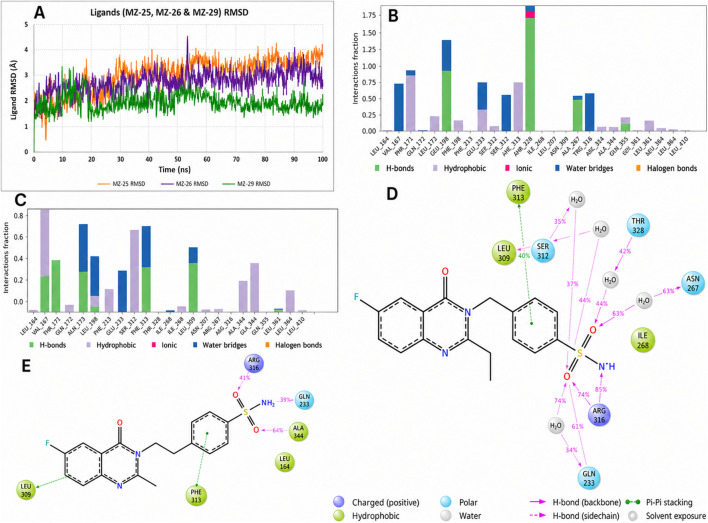
Molecular dynamics (MD) simulation analysis of MZ-29, MZ-26, and MZ-25 in complex with PPARγ. **(A)** Ligand RMSD comparison over 100 ns, showing MZ-29 (green) and MZ-26 (purple) maintaining low RMSD values (≈1–1.5 Å), indicative of stable and well-defined binding modes, whereas MZ-25 (orange) exhibits a progressive increase in RMSD to ∼4 Å, reflecting reduced dynamic stability. **(B)** Protein–ligand interaction occupancy for MZ-29, expressed as the fraction of simulation time during which key contacts are maintained, highlights persistent hydrogen bonding with Gln275 and Ser289 and frequent aromatic interactions with Phe313. **(C)** Corresponding interaction occupancy profile for MZ-26, showing a similar but slightly less persistent interaction pattern, with sustained hydrogen bonding to Gln275 and Ala327 and moderate hydrophobic contacts within the binding pocket. **(D)** MD-derived ligand–protein interaction diagram for MZ-29, illustrating the network of stable hydrogen bonds, π–π stacking, and water-mediated contacts retained throughout the trajectory. **(E)** Ligand–protein interaction diagram for MZ-25, demonstrating fewer and less persistent contacts, consistent with its higher RMSD and weaker binding stability. Collectively, the MD data identify MZ-29 and MZ-26 as the most dynamically stable ligands in the series, whereas MZ-25 shows diminished interaction persistence under simulation conditions.

The MZ–PPARγ complexes displayed intermediate dynamic stability relative to the two reference ligands. MZ-13, MZ-26, and MZ-29 exhibited consistently stable binding behaviour throughout the 100 ns simulations (Figures 11A,B; Figure 12A). MZ-29 maintained an exceptionally low ligand RMSD (≈1 Å or below) across the entire trajectory (Figure 12A), indicative of a highly constrained and well-defined binding mode. MZ-26 showed a similarly stable profile, with ligand RMSD remaining below ∼1.5 Å and minimal conformational drift, closely overlapping the stability observed for MZ-29. MZ-13 also demonstrated sustained stability, with ligand RMSD values of ∼1–2 Å and no abrupt rearrangements during the simulation.

The enhanced stability of these three ligands is supported by persistent interaction networks observed in the MD contact analyses (Figures 11B–D), including sustained hydrogen bonding to Gln275 (occupancy >90% over 100 ns) and conserved hydrophobic and aromatic interactions involving Phe313 and neighboring residues. These interactions collectively restrain ligand motion within the binding pocket and stabilize the bound conformations. In contrast, MZ-20 and MZ-25 exhibited higher ligand RMSDs and reduced interaction persistence. MZ-20 showed moderate fluctuations around ∼2–3 Å, with a gradual increase to ∼3.5 Å toward the end of the trajectory (Figure 11C), consistent with progressive loosening within the pocket and intermittent loss of key contacts. MZ-25 displayed the least stable behaviour: after approximately 50 ns, the ligand RMSD increased steadily and stabilized around ∼4 Å (Figure 11D), corresponding to a shifted binding orientation in which the sulfonylurea moiety no longer consistently engaged Gln275, as reflected by the diminished contact occupancy in the MD interaction analysis (Figure 12E). These MD-derived trends align closely with the MM-GBSA binding free energy estimates, in which MZ-25 was predicted to bind weakly. Collectively, the simulations reinforce that MZ-13, MZ-26, and MZ-29 maintain stable, well-anchored binding modes under dynamic conditions, whereas MZ-20 and particularly MZ-25 are less effective at sustaining key stabilizing interactions over time.

Quantitative analysis of MD-derived interaction occupancies further clarifies the observed differences in binding stability. In the more stable complexes—rosiglitazone, MZ-29, MZ-26, and MZ-13—the hydrogen bond to Gln275 is maintained for the vast majority of the simulation (>90% of frames), serving as a key anchoring interaction. These complexes also display persistent hydrophobic contacts within the binding pocket; notably, Phe313 engages the ligands through aromatic stacking or edge-to-face interactions for approximately 30%–50% of the trajectory, while Ile326 and Met364 frequently form a hydrophobic cradle that stabilizes the ligand core. Arg316, which can participate in hydrogen bonding or salt-bridge formation with acidic ligand functionalities, is engaged by rosiglitazone via its thiazolidinedione head group for ∼40% of the simulation and by the carboxylate moiety of MZ-29 for ∼45% of the trajectory (Figure 12C). In contrast, Arg316 shows minimal interaction with glibenclamide or MZ-25, both of which lack an appropriately positioned acidic group, highlighting a missed stabilizing interaction in these complexes. The less stable ligands (glibenclamide, MZ-25, and MZ-20) are characterized not only by reduced hydrogen-bond occupancies—most notably a Gln275 interaction present in <50% of frames for MZ-25—but also by more frequent insertion of water molecules between the ligand and protein, leading to intermittent disruption of key contacts. For glibenclamide in particular, MD trajectory inspection confirmed that following loss of the Gln275 and Ala327 hydrogen bonds, the ligand remains associated with the pocket primarily through weak hydrophobic interactions and sporadic π-stacking with Phe313 (∼10% occupancy). This limited interaction network underscores glibenclamide’s inability to adopt a tightly locked binding mode within the PPARγ ligand-binding domain, consistent with its reported weak or partial agonistic activity *in vitro*.

#### 2.6.4 ADMET properties

An essential consideration for any drug candidate is its pharmacokinetic profile. The *in silico* ADMET predictions for the MZ compounds, glibenclamide, and rosiglitazone are summarized in Table 6. Gratifyingly, all five MZ compounds exhibit zero Lipinski rule-of-five violations and had “#stars” = 0, indicating that none of their calculated properties fall outside the range of 95% of known oral drugs. Their molecular weights (329–378 Da) and calculated log P (1.16–1.80) are in a favorable range for drug-likeness. In particular, the MZ series is considerably less hydrophobic than glibenclamide (log P ∼5.7) and rosiglitazone (log P ∼5.5), which may translate to fewer formulation and off-target distribution issues. Consistent with this, the predicted aqueous solubilities (QPlogS) for MZ-13, 20, 25, 26, 29 are between −3.3 and −4.3 (approximately 20–100 µM range solubility), which, while moderately low, are far better than the extremely poor solubility predicted for glibenclamide (log S = −7.88, essentially insoluble) and also better than rosiglitazone’s solubility (predicted log S ∼ −5.97). Only glibenclamide earned 1 QikProp star, likely due to this very low solubility falling outside the drug-like range. The MZ compounds’ improved solubility is attributable to their polar sulfonylurea moiety and lower overall lipophilicity. From an absorption standpoint, the MZ compounds are predicted to have high human oral absorption (%HOA ∼73–77% absorbed). All are in the highest qualitative absorption category (grade 3, high). Glibenclamide, despite high lipophilicity, is predicted to have ∼78% absorption; however, its low solubility could make that figure optimistic. Rosiglitazone shows ∼89% absorption, consistent with its known good oral bioavailability. Importantly, the MZ compounds have calculated Caco-2 cell permeabilities in the 150–190 nm/s range, indicating moderate permeability–sufficient for absorption, though not as high as rosiglitazone’s (250 nm/s). Glibenclamide’s permeability is much lower (∼54 nm/s), likely reflecting its tendency to form insoluble aggregates; yet *in vivo* glibenclamide is absorbed (∼85%) when given in appropriate formulations, illustrating the interplay of solubility and permeability (Ntie-Kang, 2013).

**TABLE 6:** Predicted ADMET properties of glibenclamide, rosiglitazone, and the MZ compounds (MZ-13, MZ-20, MZ-25, MZ-26, MZ-29). All predictions were made using QikProp. Key properties include molecular weight (MW), partition coefficient (QPlogPo/w), aqueous solubility (QPlogS, log10 S in mol/L), predicted hERG K+ channel blocking (QPlogHERG, concerns arise if < −5.0), Caco-2 cell permeability (QPPCaco, in nm/s, >500 is excellent, <25 poor), and human oral absorption percentage. All values fall within acceptable ranges except where noted (glibenclamide’s low solubility flagged by one star, and hERG values for MZ compounds slightly below the −5 threshold).

Compound	Stars	MW (Da)	QPlogP_o/w_	QPlogS	QPlogHERG	QPPCaco (nm/s)	Oral absorption (%)
MZ-13	0	329.4	1.16	−3.33	−5.55	154	72.9
MZ-20	0	377.8	1.80	−4.30	−5.69	152	76.6
MZ-25	0	361.4	1.56	−3.96	−5.65	152	75.1
MZ-26	0	347.4	1.42	−3.68	−5.43	156	74.5
MZ-29	0	361.4	1.60	−3.97	−5.59	189	77.1
Glibenclamide	1	494.0	5.69	−7.88	−4.72	54	78.3
Rosiglitazone	1	300.4	5.49	−5.97	−2.99	250	89.1

Rosiglitazone’s properties are listed under “native ligand”; glibenclamide is also known as glyburide. The optimal range for each property (per QikProp’s recommended drug-like range) is as follows: MW, 130–725; QPlogPo/w −2 to 6.5; QPlogS −6.5 to 0.5; QPlogHERG > −5 (less negative is better); QPPCaco <25 (poor, likely not absorbed) to >500 (excellent); %Human Oral Absorption >80% = high, <25% = low. All MZ, compounds are within drug-like ranges for these parameters. Glibenclamide’s single out-of-range value is its extremely low solubility (reflected in QPlogS and marked by one star). All compounds are predicted to be CNS-inactive (CNS, category −2) and have acceptable numbers of likely metabolic routes (#metab 2 for MZs, and glibenclamide, 4 for rosiglitazone).

One area of concern highlighted by the ADMET data is the potential for hERG cardiac ion channel inhibition. The MZ compounds have QPlogHERG values around −5.5 to −5.7. Values more negative than −5.0 are often a red flag for hERG blockade liability (compounds below this threshold may bind to the hERG K+ channel and cause cardiotoxicity). All MZ compounds slightly exceed this cutoff, suggesting a possible risk of hERG inhibition that would need to be addressed or monitored in further development. In contrast, rosiglitazone’s QPlogHERG is −2.99 (no significant risk predicted) and glibenclamide’s is −4.72 (borderline but above −5). The MZ structures likely carry motifs (for example, certain aromatic or sulfonyl features) that the QikProp model associates with hERG binding. Medicinal chemistry optimization might be required to mitigate this, possibly by reducing lipophilicity further or removing specific moieties associated with hERG binding, without compromising PPARγ affinity. Aside from hERG, other ADMET parameters for the MZ series are largely unremarkable: all have low predicted brain penetration (QPlogBB ∼ −1.3 to −1.5, which is actually desirable for a diabetes drug to avoid CNS side effects). They also have acceptable predicted hepatocyte stability, with #metab = 2 likely sites of metabolism (phase I metabolism likely on the aromatic rings), which is within a normal range (glibenclamide and rosiglitazone by comparison have #metab of 2 and 4, respectively, rosiglitazone being more extensively metabolized *in vivo*). All compounds show moderate affinity for plasma proteins (QPlogKhsa ∼ −0.3 to −0.4 for MZs, 0.79 for glibenclamide, 0.82 for rosiglitazone; the slightly higher values for glibenclamide/rosiglitazone indicate more extensive albumin binding, which correlates with their high lipophilicity). High plasma protein binding can reduce free drug concentrations, but for highly potent drugs, it may not impede efficacy significantly. The MZ compounds’ moderate protein binding might actually be beneficial, ensuring a higher fraction of free drug available for action.

In summary, the ADMET predictions portray the MZ series as drug-like and orally bioavailable, with advantages over glibenclamide in solubility and over rosiglitazone in polarity (which could reduce off-target accumulation). The only cautionary finding is the uniformly predicted hERG inhibition potential, which suggests the need for further optimization to improve cardiac safety. Nevertheless, from a pharmacokinetic perspective, the MZ compounds appear suitable for progression, supporting the validity of our design strategy of combining sulfonylurea and PPARγ agonist pharmacophores.

In conclusion, in this work, we applied a comprehensive computational workflow to evaluate a series of novel sulfonylurea-based compounds (MZ-13, MZ-20, MZ-25, MZ-26, MZ-29) as dual-target antidiabetic agents, with a focus on PPARγ agonism. Using molecular docking, induced-fit modeling, MM-GBSA binding energy calculations, and 100-ns molecular dynamics simulations, we compared the behavior of these compounds to the known PPARγ full agonist rosiglitazone and the sulfonylurea drug glibenclamide. The results consistently identify MZ-29 and MZ-26 as the most promising candidates in the series. These two compounds achieved docking scores and binding orientations that closely mimicked the native ligand, including conserved hydrogen-bonding to Gln275 and favorable interactions in the receptor’s activation region. Their simulated binding free energies (ΔGbind ∼ −48 to −49 kcal/mol) were the most favorable of the series and within roughly 60% of rosiglitazone’s binding strength–a remarkable result for initial designs. MD simulations confirmed that MZ-29 and MZ-26 form stable complexes with PPARγ, maintaining key interactions over 100 ns and showing minimal ligand drift. In contrast, compounds MZ-20 and MZ-25 were less optimal: despite reasonable docking scores, they exhibited weaker binding energies and signs of partial dissociation in MD, correlating with their less robust interaction networks (e.g., failing to engage helix H12 effectively). MZ-13 performed moderately well, with stable binding in MD and good interactions, and might serve as a useful scaffold for further optimization given its smaller size.

Glibenclamide, which was included as a reference sulfonylurea, displayed only a shallow and transient binding to PPARγ. While induced-fit docking suggested that glibenclamide can adopt a pose in the pocket (anchored by the sulfonylurea–Gln275 H-bond), its MM-GBSA energy was unfavorable, and the ligand ultimately drifted in simulation. This aligns with experimental observations that glibenclamide has only weak partial PPARγ agonist activity (∼20% of a full agonist). The implication is that mere presence of a sulfonylurea moiety is not sufficient for strong PPARγ activation; additional structural features (as in the MZ compounds) are necessary to engage the receptor’s critical activation contacts.

From an ADMET perspective, the MZ compounds demonstrated drug-like profiles superior in some properties to the reference drugs. All five MZ candidates are within ideal ranges for molecular weight, lipophilicity, and polarity, with no significant flags except a uniformly modest risk of hERG inhibition that will require attention. They are predicted to be orally bioavailable (high permeability and absorption) and not prone to CNS penetration–desirable attributes for an antidiabetic therapy to avoid central effects. Notably, the MZ series’ solubilities are predicted to be significantly better than those of glibenclamide, which could translate to improved formulation and absorption consistency. The predicted pharmacokinetic profiles, therefore, support the viability of these molecules as orally administered agents.

Our integrated computational analysis strongly suggests that MZ-29 and MZ-26 emerge as lead candidates worthy of experimental validation. They combine favorable binding to PPARγ (approaching that of the full agonist rosiglitazone) with predicted pharmacokinetic properties suitable for drug development. MZ-13 also holds interest as a slightly smaller analog with good stability, potentially offering a different balance of partial agonism and reduced side effects. The dual activity concept–incorporating a sulfonylurea functionality for insulin release (SUR1 activation) with a PPARγ agonist motif for insulin sensitization–is supported by our findings, as the MZ compounds appear to retain drug-like character while engaging PPARγ in a meaningful way. Further optimization may focus on reducing the hERG liability and enhancing specific contacts with the PPARγ activation helix to boost potency. Overall, this study provides a rational basis for advancing the top MZ compounds into synthesis and biological testing. If their *in vitro* and *in vivo* profiles corroborate the computational predictions, these molecules could represent a new class of orally active antidiabetic agents that address multiple facets of type 2 diabetes by concurrently stimulating insulin secretion and improving insulin sensitivity. Such dual-action therapeutics, exemplified here by MZ-29 and MZ-26, hold the promise of improved glycemic control and patient outcomes.

### 2.7 Structural-activity relationship

1. Compounds containing a fluoride atom at the C6 position, like MZ-29 and MZ-26, have higher activity than those containing a chloride atom at the same position, like MZ-20
2. The unsubstituted molecule (MZ-13) has lower activity than the chloro or fluoro substituted
3. Compounds with an ethyl side chain at the C2 position (MZ-29) have higher activity than those with a methyl side chain (MZ-26, MZ-13, MZ-20, MZ-25)
4. The length of the linker between the quinazoline and the sulfonamide ring affects the activity. When the linker contains one carbon atom (CH2), it gives higher activity than two carbon atoms (CH2-CH2-)
5. Compounds having one carbon linker (MZ-26, MZ-29) had a longer duration of action than those that have a linker with two carbons (MZ-13, MZ-20, MZ-25).

## 3 Conclusion

New molecules of substituted quinazoline-sulfonamides were designed, synthesized, and biologically evaluated as antidiabetic agents. These molecules are structurally similar to the well-known antidiabetic glibenclamide. We performed the *in vivo* screening using a streptozotocin (STZ)-induced mouse model of T2D, with glibenclamide as the standard reference drug, in addition to the *in vitro* screening to measure the activity of these molecules against the PPARγ enzyme, and total antioxidant activity. *In vivo* screening at a dose of 2 mg/kg revealed promising activity for MZ-29, which exceeded the reference glibenclamide. The enzymatic assay and the total antioxidant activity of these molecules displayed superior or comparable activity to the reference glibenclamide. The physicochemical analysis confirmed the drug-like properties of these molecules. Furthermore, molecular modeling studies identified the highest active compounds, MZ-26 and MZ-29, as promising molecules having binding interactions like the native ligand. Consequently, these compounds represent promising candidates for further optimization and investigation as potential antidiabetic agents.

## 4 Experimental

### 4.1 Chemistry

#### 4.1.1 General procedure for synthesis of 2-methyl-4H-benzo[d][1,3]oxazin-4-one derivatives (2a-2c)

The compounds 2a-c were prepared following the reported procedures (El-Zahabi et al., 2022; Ibrahim et al., 2017a; Ramsey et al., 2007). The appropriate acid 1 derivative was refluxed in anhydrous acetic anhydride for 1–3 h. After the reaction was complete, the solvent was evaporated under vacuum, yielding 100% of the corresponding benzoxazinone intermediates.

#### 4.1.2 MZ-13: 4-((2-Methyl-4-oxoquinazolin-3(4H)-yl)methyl)benzenesulfonamide (4a)

To a solution of 2a (2.35 g) and freshly fused sodium sulfate (1 eq) in glacial acetic acid, 4-(aminomethyl)benzenesulfonamide 3a (2 eq). After refluxing for 24 h, a solid precipitated in the reaction flask as it cooled to RT and was vacuum filtered, then heated in methanol. The reaction yielded 2.23 g (46.4%) of 4a white solid. Mp: 228.6 °C–229 °C. ^1^H NMR (80 MHz, DMSO-d6) δ 8.16 (d, J = 5.9 Hz, 1H), 7.83 (d, J = 8.3 Hz, 3H), 7.72–7.49 (m, 2H), 7.42 (d, J = 6.3 Hz, 4H), 5.45 (s, 2H), 1.93 (s, 2H). ^13^C NMR (20 MHz, DMSO-d6) δ 172.41, 161.77, 155.29, 147.42, 143.43, 140.84, 134.94, 127.10, 126.99, 126.88, 126.76, 126.54, 120.16, 46.70, 23.30, 21.41. LCMS: m/z 330.3, RT = 2.17 min.

#### 4.1.3 MZ-20: 4-(2-(6-chloro-2-methyl-4-oxoquinazolin-3(4H)-yl)ethyl)benzenesulfonamide (4d)

The compound was synthesized by reacting 2c (1.14 g) with 4-(2-aminoethyl)benzenesulfonamide 4c (2 eq) following the method for 4a, and the solid was crystallized from methanol. The reaction yielded 0.833 g (37.8%) of 4d as a beige solid. Mp: 235.9 °C–237 °C. ^1^H NMR (80 MHz, DMSO-d6) δ 8.05 (d, J = 2.4 Hz, 1H), 7.78 (dt, J = 8.9, 5.9 Hz, 3H), 7.59–7.20 (m, 4H), 4.24 (t, J = 7.9 Hz, 2H), 3.04 (t, J = 7.8 Hz, 2H), 2.53 (s, 3H). ^13^C NMR (20 MHz) δ 160.21, 155.57, 145.74, 142.51 (d, J = 3.7 Hz), 134.52, 130.55, 129.15 (d, J = 10.1 Hz), 125.55 (d, J = 18.2 Hz), 121.16, 45.49, 33.28, 22.83. LCMS: m/z 378, RT = 3.11 min.

#### 4.1.4 MZ-25: 4-(2-(6-fluoro-2-methyl-4-oxoquinazolin-3(4H)-yl)ethyl)benzenesulfonamide (4c)

The compound was synthesized by reacting 2b (1.8 g) with 4-(2-aminoethyl)benzenesulfonamide 3b (2 eq) following the method for 4a. The reaction yielded 2.65 g (73.4%) of 4c as a white solid. Mp: >250 °C. ^1^H NMR (80 MHz, DMSO-d6) δ 7.97–7.67 (m, 4H), 7.67–7.41 (m, 3H), 7.37 (s, 2H), 4.43–4.08 (m, 2H), 3.07 (t, J = 7.9 Hz, 2H), 2.55 (s, 3H). ^13^C NMR (20 MHz, DMSO-d6) ^13^C NMR (20 MHz, DMSO-d6) δ 165.83, 160.46, 154.46, 143.90, 142.63, 142.49, 129.69, 129.40, 126.01, 123.55, 120.87, 111.23, 110.09, 45.42, 33.33, 22.69. LCMS: m/z 362, RT = 2.72 min.

#### 4.1.5 MZ-26: 4-((6-fluoro-2-methyl-4-oxoquinazolin-3(4H)-yl)methyl)benzenesulfonamide (4b)

To a solution of 2b (1.15 g) and freshly fused sodium sulfate (1 eq) in 12 mL glacial acetic acid, 4-(aminomethyl)benzenesulfonamide 3a (2 eq). The reaction was heated in a microwave for 3 h at 115 °C. The solid precipitate was vacuum filtered, then crystallized from methanol. The reaction yielded 0.685 g (30.6%) of 4b as a white solid. Mp 230.5 °C–232.9 °C. ^1^H NMR (80 MHz, DMSO-d_6_) δ 7.84 (d, J = 3.2 Hz, 1H), 7.76 (s, 2H), 7.68 (d, J = 6.5 Hz, 2H), 7.44 (s, 2H), 7.36 (d, J = 3.2 Hz, 2H), 5.43 (s, 2H), 2.46 (s, 2H). ^13^C NMR (20 MHz, DMSO-d_6_) δ 160.88, 154.56, 144.04, 143.24, 140.40, 129.86, 129.44, 126.92, 126.33, 123.80, 122.61, 121.30, 120.87, 111.59, 110.42, 46.62, 22.92. LCMS: m/z 348, RT = 2.72 min.

#### 4.1.6 General procedure for synthesis of 2-ethyl-4H-benzo[d][1,3]oxazin-4-one derivatives (5a & 5b)

The compounds 5a and 5b were prepared following the reported procedures (Schmidt, 2018; Sherman et al., 2006). A solution of the appropriate acid 1 and propionyl chloride (1.1 eq) was stirred in dry pyridine at RT for 2 h. After reaction completion, pyridine was evaporated, and the benzoxazinone intermediates were used for the next step without further workup.

#### 4.1.7 MZ-29: 4-((2-ethyl-6-fluoro-4-oxoquinazolin-3(4H)-yl)methyl)benzenesulfonamide (6a)

Intermediate 5a (2.07 g) was refluxed with 4-(aminomethyl)benzenesulfonamide HCl 3a (2 eq) following the procedure for 4a. The reaction yielded 1.5 g (38.9%) of 6a as a white solid. Mp: 243.5 °C–245.5 °C. ^1^H NMR (80 MHz, DMSO-d6) δ 7.76 (t, J = 6.4 Hz, 5H), 7.39 (d, J = 4.9 Hz, 4H), 5.44 (s, 2H), 2.75 (q, J = 7.0 Hz, 2H), 1.19 (t, J = 7.1 Hz, 3H). ^13^C NMR (20 MHz, DMSO-d6) δ 161.22, 157.62, 144.16, 143.41, 140.80, 130.14 (d, J = 8.3 Hz), 127.03, 126.53, 123.96, 122.78, 46.01, 27.67, 11.00. LCMS: m/z 362, RT 3.22 min.

### 4.2 Antidiabetic activity

#### 4.2.1 *In vivo* screening

Adult male albino mice (8–10 weeks old, weighing 25–35 g) were used in this study. Animals were housed in standard polypropylene cages (5 mice per cage) under controlled environmental conditions: temperature 22 °C ± 2 °C, relative humidity 50%–60%, and a 12-h light/dark cycle. Mice had free access to a standard pellet diet and water *ad libitum*. All animals were acclimatized for 1 week before the experiment. The study protocol was approved by the Institutional Committee of FCMS (IRB Approval No.: 544/IRB/2023, dated November 15, 2023). All procedures followed the ARRIVE guidelines and the guidelines of the Committee for Control and Supervision of Experiments on Animals (CPCSEA). After overnight fasting for 12 h, diabetes was induced in mice by a single intraperitoneal injection of streptozotocin (STZ, Sigma-Aldrich, United States of America), freshly dissolved in 0.1 M citrate buffer (pH 4.5) at a dose of 55 mg/kg body weight. Non-diabetic control mice received the same volume of citrate buffer alone. To prevent fatal hypoglycemia caused by excessive pancreatic insulin release, mice were given a 5% glucose solution for 24 h following STZ injection. Diabetes was confirmed by measuring fasting blood glucose levels 72 h after STZ injection. Mice with fasting blood glucose levels ≥250 mg/dL were considered diabetic and included in the study. A total of 35 mice were used, divided into 7 groups (n = 5 per group). Group I: Normal control (non-diabetic) mice; Group II: Diabetic mice treated with glibenclamide (standard drug, 2 mg/kg); Groups III-VII: Diabetic mice treated with test compounds (2 mg/kg each). Glibenclamide and all test compounds were suspended in 0.5% carboxymethylcellulose (CMC) solution and administered orally by gavage once daily for 6 consecutive days, followed by 6 withdrawal days. The dosing volume was maintained at 5 mL/kg of body weight. Normal and diabetic control groups received an equivalent volume of vehicle (0.5% CMC) orally for the same duration. Blood samples were collected from the tail tip of overnight fasted mice on days 0 (baseline) and 6, 12 of treatment. Blood glucose levels were measured immediately using a commercial glucometer (Accu-Chek Active, Roche Diagnostics, Germany) with test strips designed for rodent blood samples.

#### 4.2.2 *In Vitro* PPARγ assay

Following the manufacturer’s instructions, the assay was done with a Rat PPAR-gamma CLIA Kit (Cat. No. RTES00433). Before use, all samples and reagents were allowed to warm up to room temperature. We made standard solutions by serially diluting them until they reached concentrations of 2000, 1000, 500, 250, 125, 62.5, 31.25, and 0 pg/mL. Then, 100 μL of standards or samples was added to the wells of the plate that had already been coated. The plate was then incubated at 37 °C for 90 min. After that, the liquid was sucked out, and 100 μL of Biotinylated Detection Antibody was added to each well. The wells were then incubated at 37 °C for an hour. After washing three times with wash buffer, 100 μL of HRP Conjugate was added, and the mixture was left at 37 °C for 30 min. After that, the plate was washed five times, and 100 μL of Substrate Mixture Solution was added. It was then left in the dark at 37 °C for 5 min. Finally, a chemiluminescence plate reader was used to quickly measure the Relative Light Units (RLU). We used a four-parameter logistic curve fit to compare the RLU values to the standard curve and figure out how much PPARγ was in the samples. We did all of the measurements twice.

#### 4.2.3 Total antioxidant capacity

The total antioxidant capacity was determined using a colorimetric kit (Cat. No. TA 25 13, Biodiagnostic, Egypt) according to the manufacturer’s protocol. Briefly, Reagent 1 (substrate, H_2_O_2_) was diluted 1000-fold with distilled water immediately before use (10 μL R1 + 10 mL d.H_2_O). Working reagent was prepared fresh by mixing equal volumes of Reagent 2 (chromogen) and Reagent 3 (enzyme-buffer). To each sample tube, 20 μL of sample was added, while 20 μL of distilled water was added to the blank tube. Subsequently, 500 μL of diluted R1 was added to all tubes, mixed well, and incubated at 37 °C for 10 min. Following incubation, 500 μL of reagent was added to each tube, mixed thoroughly, and incubated at 37 °C for an additional 5 min. The absorbance of both blank (Aв) and sample (As_a_) was read immediately against distilled water at 505 nm. The total antioxidant capacity was calculated using the formula: TAC (mM/L) = (Aв − As_a_) × 3.33. All measurements were performed in duplicate.

### 4.3 Molecular modeling

The molecular modeling study was conducted by using Schrödinger’s Protein Preparation Wizard (Maestro 14.5.131, Schrödinger, LLC, NY, 2023). All details are explained in the Supplementary Material S2.

### 4.4 Statistical analysis

Statistical analyses were conducted in Python 3 using the SciPy statistical package. The significance of differences among all groups was first assessed using one-way ANOVA. Following a significant ANOVA result, specific comparisons between treatment groups and the negative control were performed using Dunnett’s post-hoc test. Data are expressed as mean ± SD. The threshold for statistical significance was set at α = 0.05, with the following notation: ***p < 0.001, **p < 0.01, *p < 0.05, ns (not significant) p ≥ 0.05.

## Data Availability

The original contributions presented in the study are publicly available. This data can be found here: https://doi.org/10.6084/m9.figshare.32055228.v1.
