# Supplementary material for: Discovery of novel quinazoline-sulfonamide derivatives with promising antidiabetic activity
Source: Front Chem. 2026 Apr 29;14:1800775. doi: 10.3389/fchem.2026.1800775 (PMC13169069; doi:10.3389/fchem.2026.1800775)
Supplement: Supplementary file 1 [file DataSheet2.docx]

**4.3. Molecular modeling**

3.3.1. Protein Preparation**:** The crystal structure of the human PPARγ ligand-binding domain (LBD) was obtained from the Protein Data Bank (PDB ID: 1FM6) [41,42]. This structure is a heterodimer of PPARγ/RXRα co-crystallized with the PPARγ agonist rosiglitazone and 9-cis-retinoic acid. For our modeling, only the PPARγ LBD (chain with rosiglitazone) was retained, and all heteroatoms (including co-crystallized ligands and water beyond 5 Å from the binding site) were removed. The PPARγ structure was prepared using Schrödinger’s Protein Preparation Wizard (Maestro 14.5.131, Schrödinger, LLC, NY). Missing side-chains were added and optimized, and protonation states of ionizable residues were assigned using PROPKA at pH 7.4. Hydrogen bonding networks were optimized, and the structure was energy-minimized with restrained heavy atoms to relieve local strains. Default parameters were applied (e.g., cap termini, fill in minimal missing loops), and crystallographic water molecules beyond the first hydration shell were deleted. The prepared receptor was then subjected to a final minimization with the OPLS4 force field to ensure a relaxed starting structure.

**4.3.2. Ligand Preparation:** Five novel sulfonylurea-derived ligands (MZ-13, MZ-20, MZ-25, MZ-26, MZ-29) were evaluated alongside glibenclamide (a sulfonylurea drug) and rosiglitazone (the native PPARγ agonist ligand in 1FM6). All ligand structures were built and energy-minimized using LigPrep (Schrödinger) with the OPLS4 force field. Possible ionization states were generated at pH 7.4 ± 0.5 using Epik. Each ligand was restricted to its predominant neutral or anionic state (if applicable) expected at physiological pH. Low-energy ring conformations were sampled, and a single minimized 3D conformation per ligand was taken forward for docking studies. In the case of glibenclamide and rosiglitazone, known active conformers were used as references.

**4.3.3. Receptor Grid and Glide Docking:** A docking grid was defined at the PPARγ ligand-binding site using Glide’s Receptor Grid Generation module. The center of the grid was placed at the centroid of the co-crystallized rosiglitazone binding pose (PDB 1FM6), encompassing key active site residues (including Gln275, Arg316, Phe313, and Tyr327 in PPARγ; residue numbering per PDB structure) within a 10×10×10 Å cube. A Glide standard precision (SP) docking was first performed for an initial ranking of ligand poses [44,45]. The receptor was kept rigid during SP docking, and up to 10 poses per ligand were retained. GlideScore was used as the primary scoring function to estimate binding affinity. No additional constraints were applied in this initial docking phase, as all ligands were expected to occupy the canonical agonist-binding pocket of PPARγ.

**4.3.4. Induced Fit Docking (IFD):** To account for receptor flexibility upon ligand binding, induced fit docking was carried out using Schrödinger’s Induced Fit Docking protocol [46]. The top poses from the SP docking were subjected to IFD. In each IFD run, ligands were re-docked into a softened receptor (van der Waals scaling of 0.50 for non-polar receptor atoms within 5.0 Å of the ligand) to allow side-chain adjustments. Up to 20 initial poses were generated per ligand using Glide SP. For each pose, Prime was used to refine the protein structure: residues within 5 Å of the ligand were optimized and minimized (allowing side-chain reorientation to accommodate the ligand) [46]. The ligand was then re-docked into each induced-fit receptor conformation using Glide XP with default settings. Final poses were ranked by the IFDScore (a composite score combining the docking score and Prime energy). The highest-ranked induced-fit pose for each ligand was selected for detailed analysis. All interactions between ligands and binding site residues were analyzed using Maestro’s 2D ligand interaction diagram tool.

3.3.5. MM-GBSA Binding Energy Calculation: To estimate ligand binding free energies, the Prime MM-GBSA module (Schrödinger) was applied to the induced-fit complexes. The binding pose of each ligand obtained from IFD was minimized with the local optimization feature, and the MM-GBSA ΔG_bind_ was calculated using the OPLS4 force field and the VSGB 2.0 implicit solvent model [43]. The ΔG_bind_ was computed as: ΔG_bind_ = E_complex_ − (E_protein_ + E_ligand_), where energies (E) are the minimized molecular mechanics energies with solvation. This approach has been shown to provide a relative ranking of ligand affinities consistent with experimental trends [13]. No further entropic corrections were applied, so the ΔG_bind_ values are reported in relative terms to compare the series.

**4.3.6. Molecular Dynamics Simulation:** Molecular dynamics (MD) simulations were performed to assess the stability of the ligand–PPARγ complexes in an explicit solvent environment. Each induced-fit complex was used as a starting structure for MD. Systems were prepared with the Desmond System Builder (Schrödinger Release 2025-3). The protein–ligand complex was embedded in an orthorhombic box of TIP3P water molecules, with a 10 Å buffer between the protein surface and box edges (periodic boundary conditions). Appropriate counterions (Na^+^ or Cl^−^) were added to neutralize the system, and 0.15 M NaCl was included to mimic physiological ionic strength. The OPLS4 force field was employed for the protein and ligand parameters. After solvation, each system underwent a brief energy minimization followed by a 6-step relaxation protocol (including restrained NVT/NPT equilibration as per Desmond default relaxation routines). Production MD simulations were then carried out for 100 ns under isothermal–isobaric conditions (NPT ensemble) at 300 K and 1 atm. The Nose–Hoover thermostat and Martyna–Tobias–Klein barostat were used to maintain temperature and pressure, respectively. A 2-fs time step was applied with the RESPA integrator (bonded interactions evaluated every step, short-range nonbonded every 2 steps, and long-range electrostatics every 4 steps). Long-range electrostatics were computed with the particle-mesh Ewald method (grid spacing ~1 Å) [41]. All bonds to hydrogen were constrained using the M-SHAKE algorithm to enable the 2-fs time step. Trajectories were saved at 100 ps intervals for analysis.

**4.3.7. Trajectory Analysis:** MD trajectories were analyzed using the Desmond Simulation Event Analysis module. Root-mean-square deviation (RMSD) of the protein Cα atoms and ligand heavy atoms (relative to the starting IFD structure) was calculated over time to monitor system stability. The protein–ligand contacts and interaction fractions were analyzed over 100 ns, with hydrogen bonds defined by a distance cutoff of 3.5 Å and angle >130°, and hydrophobic contacts within 4 Å. Root-mean-square fluctuation (RMSF) per residue was also computed to identify protein regions stabilized by ligand binding. For each complex, the Simulation Interaction Diagram was generated to visualize hydrogen bond occupancies, π–π stacking, salt bridges, and water-mediated interactions between the ligand and key amino acids throughout the simulation.

**4.3.8. ADMET Prediction:** In silico ADMET properties of all ligands were predicted using QikProp v4.5 (Schrödinger) for a comprehensive pharmacokinetic and drug-likeness assessment. QikProp generates physically relevant descriptors and uses empirical models to predict ADME properties [44]. Key descriptors such as molecular weight (MW), calculated octanol–water partition coefficient (QPlogP_o/w_), aqueous solubility (QPlogS), apparent Caco-2 cell permeability (QPPCaco, in nm/s), brain–blood partitioning (QPlogBB), human serum albumin binding (QPlogKhsa), number of likely metabolic reactions (#metab), and human oral absorption percentage were obtained. The QikProp “#Stars” index was recorded, which counts how many properties fall outside the range of 95% of known drugs (0–5 stars, with 0 indicating ideal drug-like properties). The CNS activity category was also noted (predicted central nervous system penetration, −2 = inactive, +2 = active). All ADMET predictions were made for the neutral forms of the ligands at physiological pH. These in silico ADMET parameters were interpreted with reference to standard acceptable ranges provided by QikProp (Table 8).
