# Supplementary material for: Discovery of novel quinazoline-sulfonamide derivatives with promising antidiabetic activity
Source: Front Chem. 2026 Apr 29;14:1800775. doi: 10.3389/fchem.2026.1800775 (PMC13169069; doi:10.3389/fchem.2026.1800775)
Supplement: Supplementary file 2 [file DataSheet1.docx]

## 4-((2-methyl-4-oxoquinazolin-3(4H)-yl)methyl)benzenesulfonamide (4a):

## 4-((6-fluoro-2-methyl-4-oxoquinazolin-3(4H)-yl)methyl)benzenesulfonamide (4b):

## 4-(2-(6-fluoro-2-methyl-4-oxoquinazolin-3(4H)-yl)ethyl)benzenesulfonamide (4c):

## 4-(2-(6-chloro-2-methyl-4-oxoquinazolin-3(4H)-yl)ethyl)benzenesulfonamide (4d):

## 4-((2-ethyl-6-fluoro-4-oxoquinazolin-3(4H)-yl)methyl)benzenesulfonamide (6a):
